# Structure of the quaternary complex between SRP, SR, and translocon bound to the translating ribosome

**DOI:** 10.1038/ncomms15470

**Published:** 2017-05-19

**Authors:** Ahmad Jomaa, Yu-Hsien Hwang Fu, Daniel Boehringer, Marc Leibundgut, Shu-ou Shan, Nenad Ban

**Affiliations:** 1 Department of Biology, Institute of Molecular Biology and Biophysics, Otto-Stern-Weg 5, ETH, Zurich CH-8093, Switzerland; 2 Division of Chemistry and Chemical Engineering, California Institute of Technology, Pasadena, California 91125, USA

## Abstract

During co-translational protein targeting, the signal recognition particle (SRP) binds to the translating ribosome displaying the signal sequence to deliver it to the SRP receptor (SR) on the membrane, where the signal peptide is transferred to the translocon. Using electron cryo-microscopy, we have determined the structure of a quaternary complex of the translating *Escherichia coli* ribosome, the SRP–SR in the ‘activated' state and the translocon. Our structure, supported by biochemical experiments, reveals that the SRP RNA adopts a kinked and untwisted conformation to allow repositioning of the ‘activated' SRP–SR complex on the ribosome. In addition, we observe the translocon positioned through interactions with the SR in the vicinity of the ribosome exit tunnel where the signal sequence is extending beyond its hydrophobic binding groove of the SRP M domain towards the translocon. Our study provides new insights into the mechanism of signal sequence transfer from the SRP to the translocon.

## Abstract (web summary)

Membrane proteins are inserted co-transnationally through the association between ribosome, the signal recognition particle and its receptor, and the membrane-bound translocon. Here the authors present a cryo-EM reconstruction of this quaternary complex in the activated state and propose a model for signal sequence transfer to the translocon.

Membrane proteins are targeted to the membrane while being synthesized by ribosomes. In this process the emerging signal sequence is recognized by the signal recognition particle (SRP) and delivered to the membrane to interact with the SRP receptor (SR)^1^,^2^,^3^, where the nascent chain is transferred to the Sec translocon^4^,^5^. Our mechanistic understanding of co-translational protein targeting and membrane insertion is based on structures and biochemical experiments of isolated components of the machinery, as well as ribosomal complexes with either SRP and SR or the translocon^6^,^7^,^8^,^9^,^10^. In bacteria, SRP is a ribonucleoprotein complex consisting of the SRP protein (Ffh) and the 4.5S SRP RNA that forms a hairpin structure. SRP binds to ribosome-nascent chain complexes (RNCs) displaying signal sequences. RNCs are then delivered to the membrane through interaction of SRP with SR (FtsY), during which the N and GTPase domains (termed the NG domain) in both Ffh and SR form a GTP-dependent heterodimer. The initial binding of SR occurs close to the ribosomal tunnel exit and involves interactions with the tetraloop at the tip of the SRP RNA hairpin (early state)^9^,^11^,^12^,^13^. Subsequently, stimulated by the binding of translocon^14^, the NG heterodimer detaches from the tetraloop (closed state) and repositions to the distal region of the SRP RNA, where insertion of an RNA base into the GTPase active site of SRP and SR induces GTP hydrolysis (activated state)^15^,^16^,^17^. Relocation of the NG heterodimer allows the translocon to take over the signal sequence bound to the M-domain of Ffh and to get access to its binding site on the ribosomal tunnel exit.

Previous cryo-EM studies of targeting complexes from eukaryotic and bacterial systems could only resolve the SRP–SR complex in the ‘early' or the ‘closed' states. Bacterial complexes of the closed state depicted an NG heterodimer detached from the SRP RNA tetraloop in which the distal region of the SRP RNA could not be fully visualized due to flexibility^9^,^18^. Previous cryo-EM studies on the eukaryotic SRP–SR complex revealed an additional density at the SRP distal region^19^ that was recently interpreted by docking a crystal structure of the eukaryotic NG heterodimer into this density^20^,^21^,^22^.

Several studies proposed a model for the transfer of the nascent chain from the targeting to the membrane insertion machinery where the SRP, SR and the translocon could bind simultaneously on the ribosome in a transient quaternary complex to transfer the signal sequence from the SRP M-domain to the translocon^7^,^8^,^9^,^10^,^23^,^24^,^25^,^26^. According to this model, the translocon is initially recruited to the targeting complex via hydrophobic and electrostatic interactions with the intrinsically disordered A-domain of SR^23^,^24^,^27^. However, in spite of its central importance in the SRP cycle, such a quaternary complex has never been visualized and it is currently not understood how the final events of this process are spatially and temporally orchestrated. To better understand these late series of events, we assembled a quaternary complex including *E. coli* SRP, SR and the SecYEG translocon and a translating ribosome and then resolved its structure using electron cryo-microscopy (cryo-EM).

## Results

### Structure of the RNC–SRP–SR–SecYEG quaternary complex

To biochemically characterize the formation of a quaternary complex, we used a previously established *in vitro* translation system^28^ to programme ribosomes to translate a nascent chain containing the first trans-membrane helix of the FtsQ protein (RNC_FtsQ_-85), a physiological substrate for SRP mediated co-translational protein targeting^29^,^30^. Co-sedimentation assays demonstrated that in the presence of either a non-hydrolysable GTP analogue guanosine 5′-[β,γ-imido]triphosphate (GMPPNP) or a transition state analogue (GDP:AlFx), SRP, SR and SecYEG co-sediment with the RNC in stoichiometric amounts (Supplementary Fig. 1). These results confirm previous observations that a quaternary complex can be formed on the translating ribosome^23^,^24^,^31^.

In the presence of a transition state mimic (GDP:AlFx), the isolated SRP–SR was previously shown to adopt the ‘activated' state primed for GTP hydrolysis^17^. However, such complex was never visualized on the translating ribosome. To resolve the SRP–SR NG heterodimer in the activated state, we assembled a quaternary complex between the SRP, SR, SecYEG and the RNC in the presence of GDP:AlFx. To increase the stability of the complex during the freezing procedure for our cryo-EM studies, we used the RNC_1A9L_-85 construct, as it was shown to exhibit high binding affinities to SRP, SRP–SR and the Sec translocon both in biochemical^32^ and recent structural studies^9^. This signal sequence is active in protein targeting, as evidenced by stimulation of the GTPase activity of the heterodimer and formation of the quaternary complex with the Sec translocon^14^,^31^.

**Figure 1:**
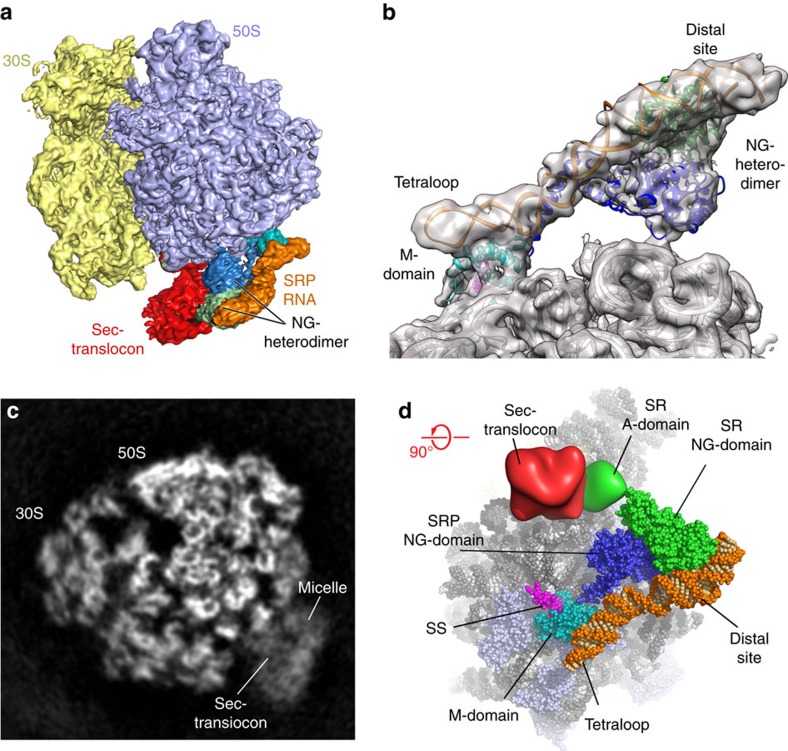
Structure of the SRP–SR–Sec translocon and RNC quaternary complex. (**a**) Cryo-EM reconstruction of the SRP–SR–Sec translocon with RNC quaternary complex at 4.8 Å (SRP RNA—orange, SRP protein M domain—cyan, SRP protein NG domain—dark blue, SR—green, translocon—red). (**b**) Representative view of the conformation of the SRP RNA and the SRP–SR NG heterodimer in the ‘activated' state, with overlaid EM density filtered to 5 Å. (**c**) Cross-section of the unsharpened EM-density map of the quaternary complex showing secondary structural elements of the Sec translocon within the unstructured detergent micelle. (**d**) The structure of the complex shown towards the ribosomal tunnel exit. Structures that were interpreted with atomic coordinates are shown as spheres, whereas, the A domain of the receptor and the Sec translocon are depicted as a surface representation. Signal sequence (SS) is shown in magenta.

The quaternary complex, comprising *E. coli* SRP, SR and the translocon assembled on a translating ribosome, was then investigated by cryo-EM. Image classification revealed that in the sample used for the cryo-EM experiment, 16% of the particles contain the quaternary complex. In this complex, the NG heterodimer is in the activated state bound to the distal site of the SRP RNA, where we observe the translocon positioned near the polypeptide exit tunnel (‘Methods' and Supplementary Fig. 2, top panel). The SRP RNA is visible in its entirety, along with fully interpretable densities for all domains of Ffh and FtsY as well as a density for the SecYEG translocon, which is anchored by the SR near the ribosome exit tunnel. In addition, 12% of the particles represented the recently visualized SRP–SR complex in the ‘early' state^9^, a conformation in which no density for the translocon was observed. To improve the resolution of the factors bound in the quaternary complex, additional image sorting and refinement were performed (Supplementary Fig. 2, bottom panel) yielding a final reconstruction resolved to 4.8 Å resolution (Fig. 1, Supplementary Fig. 3, Supplementary Table 1).

### Conformation of the SRP RNA in the activated SRP–SR complex

**Figure 2:**
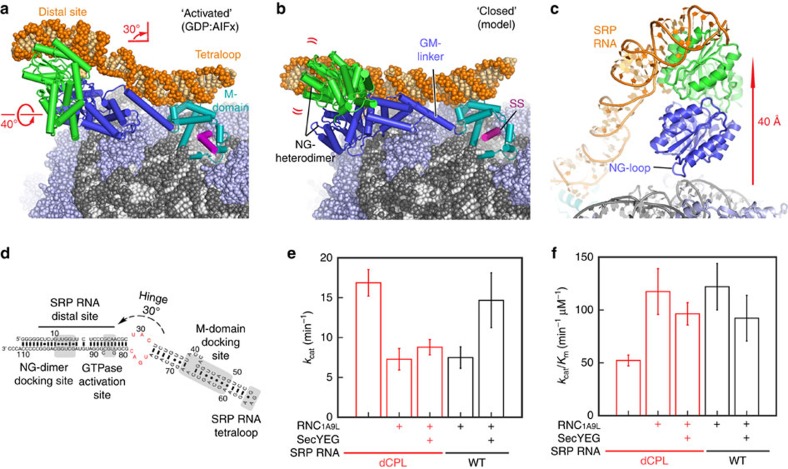
Conformation of the SRP–SR ribosome complex in the ‘activated' state. (**a**) Atomic model of the SRP–SR complex bound to the RNC in the ‘activated' state. The curved arrows demonstrate a kink upwards by 30° and an anti-clockwise untwisting by 40° in the SRP RNA, compared to **b**. (**b**) Composite model of the NG heterodimer as seen in the crystal structure of the isolated SRP–SR complex (PDB:2XXA), here shown docked on the ribosome based on the known position of the M-domain (PDB:5GAG). (**c**) Close-up view of the interaction between H100 of the rRNA and the NG loop of SRP displayed as cartoon. (**d**) Secondary structure of the *E. coli* SRP RNA: the tetraloop, M-domain, GTPase activation and NG heterodimer docking sites are highlighted in grey. The SRP RNA C-loop region, where the 30° kink in the RNA is observed, is shown in red. (**e**) Effect of the C-loop mutation of the SRP RNA on the SRP–SR GTPase hydrolysis rate (*k*_cat_) in the presence and the absence of the RNC_1A9L_-85 and the Sec translocon. (**f**) Effect of the C-loop mutation on the assembly rate constant of the SRP–SR complex (*k*_cat_/*K*_m_) with or without RNC and SecYEG present.

Previous structural studies indicated that in the presence of GMPPNP, the bacterial NG heterodimer detaches from the SRP RNA tetraloop and the distal region of the RNA becomes disordered (closed state; Fig. 2b and Supplementary Fig. 4)^9^,^18^. Notably, densities for the NG heterodimer or the Sec translocon were not observed in these complexes. In the presence of the GDP:AlFx used in this study, we can now visualize the SRP RNA in its entire length. Starting from the tetraloop, it is kinked upward by 30° and untwisted at the distal site by a 40° rotation, adopting a distinct conformation with the distal region of the SRP RNA lifted by 40 Å away from the surface of the ribosome (Fig. 2a,c, Supplementary Movies 1 and 2). The density of the NG heterodimer at the distal site was locally resolved to ∼7–8 Å (Supplementary Fig. 3, Supplementary Table 3), which allowed us to unambiguously dock without any further adjustments the crystal structure of the GDP:AlF_4_ trapped NG heterodimer in complex with the distal region of the SRP RNA (helix 5 of domain S) (Supplementary Table 2)^17^. In addition, the density for the GM linker connecting the M and NG domains could be interpreted as an α helix, similarly as observed in the crystal structure of the isolated SRP–SR complex (Supplementary Fig. 4c and d)^15^.

Due to twisting of the SRP RNA, the NG heterodimer and the distal region of SRP RNA is rotated by 40° and positioned between the SRP RNA and the ribosome. It contacts H100 of the 23S rRNA through a loop that connects the G-domain of the SRP protein to its N-domain, which we refer to as the ‘NG loop' (Fig. 2c, Supplementary Fig. 4e). Three conserved residues in this loop (Gln^98^, Pro^99^ and Pro^99^) are in close contact with the rRNA and possibly mediate the interaction with the ribosome (Supplementary Fig. 4f). The contact area between the NG loop and the ribosome replaces the contacts between the ribosome and the SRP RNA observed in all earlier RNC–SRP targeting complexes in both bacterial and eukaryotic systems and was suggested to have a role in GTP hydrolysis^9^,^33^,^34^. The extended conformation of the SRP RNA seen in early targeting complexes^15^,^19^ would not be compatible with a conformation necessary for GTPase stimulation 17 because the NG heterodimer would clash with the ribosomal surface. However, GTPase stimulation is now possible when the SRP RNA is raised up from the ribosomal surface to accommodate the SRP–SR NG heterodimer between the RNA and the ribosome, as observed in this structure (Fig. 2a,c).

### The C-loop is essential for SRP-SR GTPase activation

The rotation and kinking of the SRP RNA occurs at structurally conserved regions termed the C- and E-loops (Supplementary Fig. 5). In bacteria, the E-loop was reported to be essential in GTPase activation^17^,^35^, and a rotation in this region was previously observed^17^,^19^,^20^. In contrast, the position of the C-loop coincides with a three-way junction present in eukaryotic SRPs, and a 20° bending of this region downwards toward the ribosome at rRNA helix H100 was observed upon binding of the SRP68 and SRP72 proteins to the SRP RNA^20^,^21^,^34^. However, the C-loop was never implicated in conformational changes that lead to GTP hydrolysis.

To test whether the ability of SRP RNA to adopt the kinked conformation at the C-loop is required for recruitment of the translocon in the activated RNC–SRP–SR complex, we designed a mutant in which this loop is closed by introducing base-pairing residues to limit the conformational flexibility of the RNA (dCPL mutant; Fig. 2d). We tested for GTPase hydrolysis rates and formation of the SRP–SR complex in the presence and absence of the RNC and the Sec translocon. The GTPase activity of the SRP–SR complex in this assay strictly correlates with movement of the SRP–SR NG heterodimer to the distal site of SRP RNA in single-molecule analyses^16^,^35^. As previously reported^31^, the GTPase rate of the wildtype SRP–SR complex was reduced by the RNC and re-activated upon addition of the SecYEG complex (Fig. 2e). In contrast, closing the C-loop of the SRP RNA abolished GTPase stimulation exhibited by the translocon (Fig. 2e, red). A control experiment revealed that the dCPL mutation had no effect on the assembly rate of the SRP–SR complex, which is reflected by the value of *k*_cat_/*K*_m_ in the stimulated GTPase reaction of SRP with SR (Fig. 2f). This further corroborates that the loss of GTPase activity in the dCPL mutant is a direct consequence of the reduced flexibility of the SRP RNA, which prevents the distal site from accommodating the NG heterodimer. These results, in combination with our structural observations, suggest a mechanism where the conformational change in the C-loop of the SRP RNA is required for the recruitment of the translocon to activate the SRP–SR GTPases in the quaternary complex.

### SecYEG is positioned by the SR adjacent to the tunnel exit

**Figure 3:**
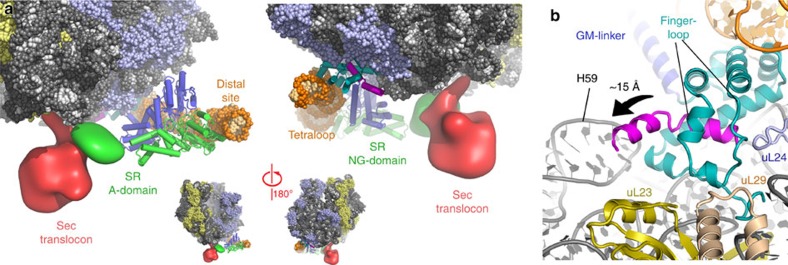
Localization of the translocon and the signal sequence in the quaternary complex. (**a**) Position of the Sec translocon on the RNC in close proximity to the extending signal sequence, bound to the SRP M domain, and the A-domain of SR. (**b**) Close-up views of the interactions between the M-domain fingerloop of the SRP protein and the ribosomal protein uL29 (wheat), where the signal sequence extends three helical turns outwards from the M-domain.

In our quaternary structure, we also observe an additional disk-shaped density connected to the SR that resides ∼30 Å from the SRP M-domain and the ribosome exit tunnel. Local resolution estimation of this region indicates that this density was resolved at 7–12 Å (Supplementary Fig. 3, Supplementary Table 3) revealing the funnel shape features of the translocon^36^,^37^,^38^ within the detergent micelle. Due to the limited resolution of this region, we can only obtain a tentative fit of the Sec translocon based on the X-ray structure of the translocon from *Thermus thermophilus*^39^ using the cytoplasmic loops (loop 6/7 and loop 8/9) as a guide (Supplementary Fig. 6, Supplementary Movie 2, Supplementary Table 2). Although the conformation of the SRP–SR at the distal site is compatible with the membrane plane (Supplementary Fig. 6d), the translocon would have to tilt by ∼20° relative to its position to be accommodated in the membrane environment as observed in the native non-solubilized state^40^ or in nano-discs^41^. In our quaternary complex, we also observe the translocon in contact with the ribosome (Fig. 3a). Although the functional relevance of these interactions is currently not clear, it is possible that they stabilize the activated state of the SRP–SR complex in agreement with the observation that the translocon stimulates their GTPase activity (Fig. 2e).

The bound translocon is surrounded by the density that can be attributed to the detergent micelle; however, we also observe a region of stronger density connecting it to the NG domain of the SR (Supplementary Fig. 7). Although in this area the secondary structure elements are not resolved, we assigned this density to the SR A-domain since previous studies showed that the A-domain binds to both the membrane and the translocon^23^,^24^,^42^,^43^,^44^,^45^. Furthermore, such a positioning of the A-domain between the translocon and the NG domain of the SR is consistent with recent biochemical results, which indicate that the translocon activates the receptor by binding to the A-domain and separating it from the NG domain^27^.

### The positioning of SecYEG relative to the signal sequence

**Figure 4:**
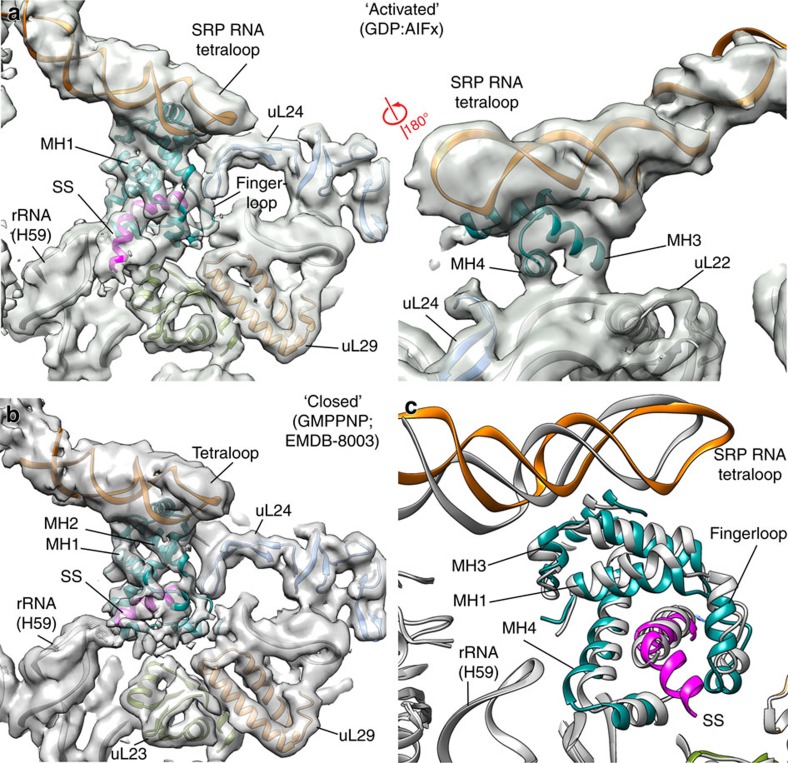
Conformational state of the M-domain. (**a**) EM densities of the quaternary complex in the pre-transfer state (this study) with fitted atomic coordinates. (**b**) EM density of the closed state of the SRP–SR complex solved in the presence of GMPPNP (EMDB:8003, PDB:5GAG), filtered to 5 Å. (**c**) Overlay the two atomic coordinates to illustrate the conformational change. Atomic coordinates of the M-domain and the signal sequence in the ‘closed' state are shown in grey. Colouring scheme is identical to Fig. 1.

The density in the vicinity of the ribosomal tunnel exit is resolved to ∼5–6 Å resolution (Supplementary Fig. 3f, Supplementary Table 3) and can be interpreted by fitting high-resolution structures of the M-domain in complex with helix 8 and the tetraloop of the 4.5S SRP RNA (Supplementary Table 2, Supplementary Movie 4)^9^,^46^,^47^. Notably, the signal sequence in this complex extends 15 Å from the SRP M-domain towards the Sec translocon along the surface of the ribosome in the vicinity of H59 of the rRNA (Fig. 3, Supplementary Movie 5). Such an extension of the signal sequence was not observed in the closed state complex (Fig. 4a,b) or in structural studies of complexes corresponding to earlier stages of the SRP targeting cycle from bacterial or eukaryotic systems^9^,^18^,^33^,^34^,^48^,^49^. Due to the conformational change in the SRP RNA, which is kinked upwards on the distal site and acts as a lever, we observe that the M-domain is now raised on one side, in contrast to its position in the RNC–SRP–SR complex in the closed state (Fig. 4c). This observed conformation of the M-domain is possibly required for reducing the affinity of SRP and its subsequent replacement by the translocon on the surface of the ribosome.

Previous studies have reported that SRP binding on the ribosome and the rate of signal sequence transfer to the translocon is dependent on the length of the nascent chain^50^,^51^,^52^,^53^,^54^. Considering that the shorter nascent chains used here to trap the quaternary complex lead to transfer of the signal sequence at a slower rate^14^,^55^, it is possible that the positioning of the translocon in our reconstruction corresponds to the cargo ‘pre-transfer' state, where the inactive translocon awaits the extending signal sequence. Continued protein synthesis will increase the length of the nascent chain, eventually allowing it to bind to the translocon. Nascent chain handover would then be followed by repositioning of the translocon to the ribosomal tunnel exit and displacement of the M-domain.

To provide experimental evidence for this sequence of events, we investigated the effect of a 50 amino acids longer nascent chain (RNC_1A9L_-135)^14^ on the formation of a quaternary complex by performing cryo-EM analysis of the RNC in the presence of SRP, SR, the translocon and GDP:AlFx. We observe that the translocon is predominantly accommodated at the ribosomal tunnel in this complex (Supplementary Fig. 8), whereas no such class was observed in the data set containing the quaternary complex (Supplementary Fig. 2). In combination with biochemical data^14^, our structures with nascent chains of different length suggest that the conformation of the quaternary complex observed here corresponds to a ‘pre-transfer' state.

## Discussion

**Figure 5:**
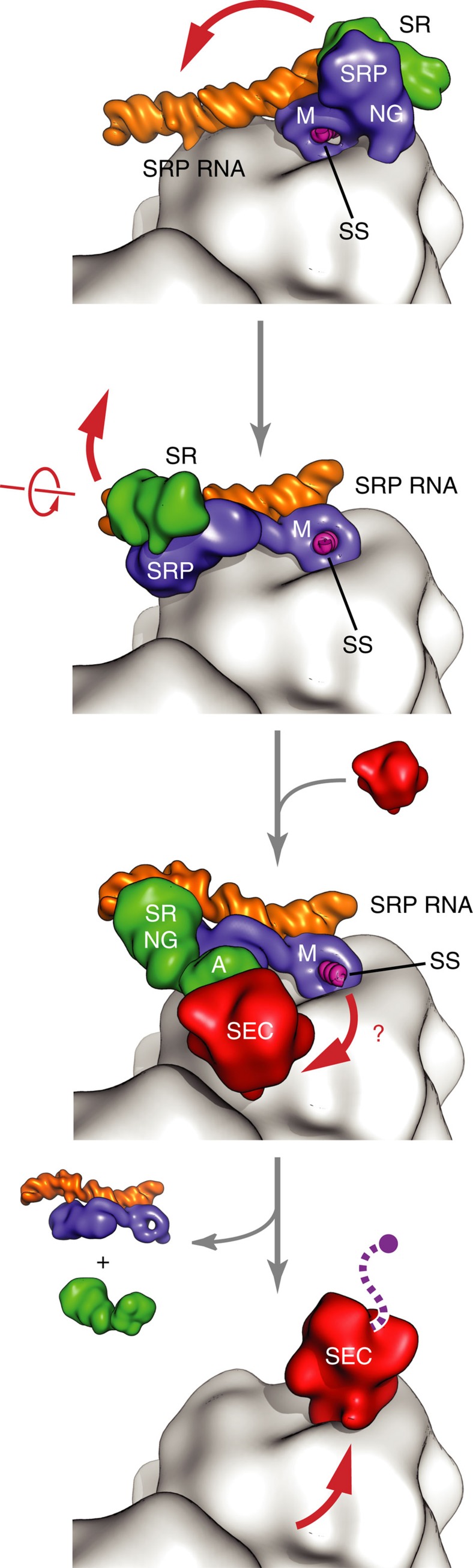
Proposed cargo handover mechanism of co-translational protein targeting. Schematic of the early, closed and activated/quaternary targeting complexes ending with the RNC in complex with the Sec translocon. Initially the SR binds to the SRP RNC complex at the tetraloop side of the SRP RNA, followed by repositioning and docking of the NG heterodimer to the distal region of SRP RNA. GTPase activation becomes possible upon conformational change in the SRP RNA that accommodates NG heterodimer between the SRP RNA and the ribosomal surface. Signal sequence handover is favored for longer nascent chains that can reach Sec translocon. Sec translocon will then displace the M-domain and SRP on the ribosome exit tunnel to complete the targeting cycle.

The structure of the quaternary ‘pre-transfer' complex provides a framework for a better understanding of the process of signal sequence handover. We show that the SRP RNA adopts a kinked and untwisted conformation to accommodate the ‘activated' state of the SRP–SR NG heterodimer at the distal site and that this conformation is required for the stimulation of GTP hydrolysis to complete the targeting process (Fig. 5). In addition, we visualize a quaternary complex where the translocon is anchored *via* the SR, prior to cargo handover. The signal sequence is observed extending beyond its binding pocket in the direction of the recruited translocon. We also show that longer nascent chains will capture the translocon and increase the rate of its accommodation at the tunnel exit, where a displacement of the M-domain would occur as proposed previously^7^,^8^,^9^,^10^,^19^,^23^,^24^,^25^,^26^. These results, combined with previous structural and biochemical work, now provide a nearly complete overview of the functional states that take place during the process of co-translational membrane protein targeting and insertion.

## Methods

### Protein purification

pET24aFfh and pUC19Ffs were co-transformed into *E. coli* strain BL21Star(DE3) (Invitrogen). Cells were induced with 1 mM isopropyl β-D-1-thiogalactopyranoside (IPTG) for 3 h at 37 °C, and then lysed using a French press. The lysate was cleared for 15 min twice at 30,000*g* using a Sorvall centrifuge. SRP was captured from the cleared cell lysate via His-Trap column (GE Healthcare), followed by ion exchange (MonoQ, GE Healthcare) over a 20–40% gradient of 1 M KCl in Buffer A (50 mM HEPES-KOH, 100 mM KCl, 10 mM MgCl_2_, 1 mM TCEP, 5% glycerol, pH 8.0) followed by size exclusion (S200, GE Healthcare) chromatography. FtsY was expressed from a pET24aFtsY vector using a similar purification procedure as described for the SRP and stored in Buffer B (50 mM HEPES-KOH, 100 mM KCl, 10 mM MgCl_2_, 5% glycerol, pH 7.3). Bacterial translocon (SecYEG) was purified from BL21C43 (DE3) cells transformed with pTrc99a_SecYEG with 1 mM IPTG at 18 °C for 16 h. Cell membranes were solubilized for 1 h using a final concentration of 1% n-dodecyl β-D-maltoside (DDM) for a 10 mg ml^−1^ protein solution. Purification of the solubilized membranes was carried out using His-Trap, SP Sepharose, and S200 columns (GE Healthcare) in Buffer B and a final concentration of 0.02% of DDM. All samples were concentrated and frozen at −80 °C until further use.

### Preparation of RNC complexes

RNC complexes were prepared using an *in vitro* translation system prepared in house as previously described^28^. RNCs were stalled using mRNA harbouring a SecM stalling sequence and containing the first trans-membrane helix of FtsQ^28^ or an engineered signal sequence (1A9L; 85 or 135 amino acids) based on the PhoA protein^32^ followed by an N-terminal 3 × Strep-tag sequence. All samples were flash frozen at −80 °C, until further use. All purification procedures were performed at 4 °C unless stated otherwise.

### Formation of the quaternary complex

RNC_FtsQ_-85 (500 nM) were incubated with the SRP, SR and SecYEG by adding first SRP and later SR and SecYEG (1:5:5:5 molar ratio) in the presence of either GMPPNP or GDP:AlFx (refs 17, 56). The reactions were incubated at 25 °C for 30 min in Buffer B without glycerol, and 0.02% DDM was added together with the translocon. The reactions were cooled on ice and then overlaid on 40% sucrose cushion (w/v) and spun for 3 h at 90,000 r.p.m. using a TLA-100 ultracentrifuge rotor (Beckman Coulter). Pellets were suspended in Buffer B, analysed with 12% SDS–polyacrylamide gel electrophoresis (PAGE) and stained with Comassie brilliant blue. For cryo-EM studies, the reaction mix containing RNC_1A9L_-85 or -135 (250 nM) was incubated with SRP, SR and the SecYEG translocon as described above using cryo-EM Buffer C (50 mM HEPES-KOH, 85 mM KOAc, 15 mM Mg(OAc)_2,_ 0.02% DDM, 5 mM spermidine, 0.5 mM spermine) in the presence of GDP:AlFx. To minimize sample background during cryo-EM data collection, we used molar ratios of 1:1:3:8 (SRP:SR:SecYEG). The final concentration of SecYEG was maintained at its calculated *K*d (∼2 μM) as reported earlier for the quaternary complex^14^,^31^.

### Data collection

The reaction mix was applied directly on Quantifoil Holey Carbon grids, which had been pre-coated with a fresh thin layer of carbon and glow-discharged for 30 s. The grids with applied sample were incubated for 1–2 min before blotting for 8 s with a FEI vitrobot at 100% relative humidity and temperature at 6 °C. Blotted grids were plunge frozen in liquid ethane cooled to liquid nitrogen temperature. Data was collected on a Titan Krios cryo-transmission electron microscope (FEI Company) operated at 300 keV and equipped with a Falcon II direct electron detector. The EPU software was used for data collection within a defocus range of −1.2 to −3.6 μm at × 100,719 magnification. A total of seven frames were collected for each image with a total dosage of 20 electrons per Å^2^. The movie frames were aligned using DOSEFGPU DRIFTCORR^57^ to correct for beam-induced movement.

### Structure calculation

Initially, the aligned frames were inspected for ice quality and cracks in carbon, the CTF was estimated using CTFFIND3 (ref. 58), and only frames with power spectra extending beyond 5 Å resolution were kept. A total of 1.02 million particles were automatically picked using Batchboxer implemented in EMAN^59^. Two-dimensional (2D) classification was performed on binned images using the maximum-likelihood refinement algorithm implemented in RELION^60^ to select for 2D averages exhibiting high-resolution features (∼790 K good particles were retained). After 2D classification, three-dimensional (3D) refinement was performed using as a reference an empty 70S ribosome low-pass filtered to 60 Å on binned images (Supplementary Fig. 2). Using the ‘skip-align' option in RELION, focused 3D classification was performed for the area around the polypeptide exit tunnel. Two classes were selected with features displaying the NG heterodimer bound either at the tetraloop (∼12% of the particles) or at the distal site of the SRP RNA, the latter also showing a density of a micelle-embedded translocon (16% of the particles), depicting the quaternary complex. Note that no class containing RNC in complex with the translocon alone was detected. The first class was refined to 3.9 Å resolution and represented the ‘early' state complex, which was recently described^9^ and is not further discussed here. The second class represented the quaternary complex and displayed a kinked SRP RNA and densities for the SRP–SR and Sec translocon, and was refined to 3.8 Å resolution. To improve the density of the SR–SRP and the translocon, a second round of 3D classification was performed on binned images by masking out the ribosome density and focusing on the distal site region. This process was then followed by a final round of 3D classification by subtracting the signal of the ribosome as previously described^61^ (Supplementary Fig. 2). The 3D class displaying the strongest density for the NG heterodimer and for the translocon was retained and refined to 4.8 Å resolution (Supplementary Figs 2 and 3, Supplementary Table 1). The data set of the complex with the longer nascent chain (RNC_1A9L_-135) was processed similarly as described above. After 2D classification, 3D refinement was then performed using as a reference an empty 70S ribosome low-pass filtered to 60 Å on binned images (Supplementary Fig. 8). Using the ‘skip-align' option in RELION, focused 3D classification was performed for the area around the polypeptide exit tunnel. Note that no density of the SRP–SR NG heterodimer or the Sec translocon was observed at the distal region. The class displaying density for the translocon at the ribosome tunnel was refined to 4.3 Å resolution.

### Molecular docking, modelling and refinement

The local resolution of the EM map was estimated based on ResMap^62^. Coordinates of the 50S ribosomal subunit and the SRP core (PDB: 5GAG) were docked into the cryo-EM map as two rigid bodies (Supplementary Table 2). For the EM density of the NG heterodimer at the distal end in the quaternary complex, we docked the crystal structure of the NG heterodimer in complex with the distal site SRP RNA as one rigid body (PDB: 4C7O). The NG loop of SRP interacting with the ribosome was manually adjusted using the programme O^63^,^64^. The kinked region of the SRP RNA and the signal sequence extension were manually modelled using COOT^65^. Homology models for the *E. coli* SecY, SecE and SecG were generated using Phyre2 (ref. 66) based on the X-ray structure of the isolated SecYEG from *T. thermophilus* (PDB: 5CH4). The resulting model was then docked as one rigid-body into the micelle-embedded translocon density only as a tentative fit due to the limited resolution of this region (∼7–12 Å) using the cytosolic loops of SecY as a guide with no further adjustments. To eliminate side chain clashes resulting from the rigid-body docking procedure and to optimize the geometry of the newly modelled areas, the docked models and contact areas on the ribosome were subjected to geometry minimization and phase restrained reciprocal space refinement against the mlhl target using PHENIX^67^. For this procedure, phases and amplitudes were back calculated from the experimental cryo-EM map, and protein secondary structure and RNA base pair restraints were applied throughout. For the final model, only the C-alpha backbone of SecYEG model was deposited.

### GTPase assay

The SRP RNA C-loop mutant (dCPL) was constructed using the QuikChange mutagenesis protocol (Stratagene) following manufacturer's instructions. The sequence of the C-loop (75–78) was replaced (UGAC to GUA) to allow base-pairing with the opposite strand of the loop (CAU; residues 29–31). Wildtype and mutant SRP RNAs were expressed and purified as described^16^,^68^. The GTPase assay to measure the stimulated GTP hydrolysis reaction between SRP and FtsY was carried out and analysed as described^69^. The reaction mixtures were assembled with 40 nM Ffh, 100 nM of SRP RNA (wild type or dCPL), 0, 0.2, 0.5, 1, 3 and 10 μM SR, and 120 nM RNC_1A9L_-85 and 6 μM of SecYEG where applicable. Reactions were carried out in 50 mM HEPES-KOH (pH 7.5), 150 mM KOAc, 10 mM Mg(OAc)_2_, 2 mM DTT, and 0.01% Nikkol. Reactions were performed in triplicates and initiated by addition of 100 μM GTP (doped with γ-^32^P-GTP) and quenched with 0.75 M KH_2_PO_4_ (pH 3.3) at various time points. The hydrolysed phosphate and unreacted GTP were separated by thin-layer chromatography and quantified by autoradiography. The observed GTPase rate constants as a function of SR concentration were analysed to derive the values of *k*_cat_ and *k*_cat_/*K*_m_ (refs 69, 70). Note that the value of *k*_cat_/*K*_m_ in this reaction is rate limited by and hence reports on the rate of SRP–SR assembly^69^, and the value of *k*_cat_ is proportional to the fraction of SRP–SR complex that attain the activated conformation, from which rapid GTP hydrolysis occurs^16^,^35^. The errors are estimated from the deviations of the data points from the fitting values at the three saturating concentrations ([FtsY]=1, 3, 10 μM).

### Making figures and plots

All figures were made either using UCSF CHIMERA^71^ or PyMOL (The PyMOL Molecular Graphics System Version 1.7 Schrödinger, LLC.). Local resolution maps were produced using ResMap^62^.

### Data availability

Cryo-EM maps have been deposited in the Electron Microscopy Databank with accession codes EMD-3617. The coordinates of the atomic structures of the 50S ribosomal subunit in complex with SRP, SR and the Sec translocon were deposited with PDB codes 5NCO. The data that support the findings of this study are available from the corresponding author upon request.

## Additional information

**How to cite this article:** Jomaa, A. *et al*. Structure of the quaternary complex between SRP, SR, and translocon bound to the translating ribosome. *Nat. Commun.* **8,** 15470 doi: 10.1038/ncomms15470 (2017).

**Publisher's note:** Springer Nature remains neutral with regard to jurisdictional claims in published maps and institutional affiliations.

## Supplementary Material

Supplementary Information Supplementary Figures, Supplementary Tables and Supplementary References

Supplementary Movie 1 Overview of the SRP, SR, SecYEG complex bound to the translating ribosome. The 3D map was filtered to 8 Å resolution. Coloring scheme is identical to the one used in the main figures.

Supplementary Movie 2 Close-up of the activated SRP-SR NG heterodimer bound to the distal site of the SRP RNA and to the surface of the ribosome.

Supplementary Movie 3 Close-up of the proposed fit of the SecYEG translocon bound via the SR to the quaternary complex. The depicted view is a cut through the DDM detergent micelle.

Supplementary Movie 4 Close-up of the M-domain (cyan) of the SRP protein bound to the signal sequence (magenta) in the activated sta te.

Supplementary Movie 5 Morphing of the SRP core region in the closed and the activated states. In the activated state, the signal sequence extends outward beyond the M-domain binding groove by 15Å.
