# Supplementary material for: Structure of the quaternary complex between SRP, SR, and translocon bound to the translating ribosome
Source: Nat Commun. 2017 May 19;8:15470. doi: 10.1038/ncomms15470 (PMC5454536; doi:10.1038/ncomms15470)
Supplement: Supplementary Information — Supplementary Figures, Supplementary Tables and Supplementary References [file ncomms15470-s1.pdf]

**Structure of the quaternary complex between SRP, SR, and translocon bound to the translating ribosome**

Ahmad Jomaa[1], Yu-Hsien Hwang Fu[2], Daniel Boehringer[1], Marc Leibundgut[1], Shu-ou Shan[2], and Nenad Ban[1]*

[1]Department of Biology, Institute of Molecular Biology and Biophysics, Otto-Stern-Weg 5, ETH Zurich, CH-8093, Switzerland
[2]Division of Chemistry and Chemical Engineering, California Institute of Technology, Pasadena, CA 91125, USA
*Correspondence to: ban@mol.biol.ethz.ch

**Supplementary Information:**

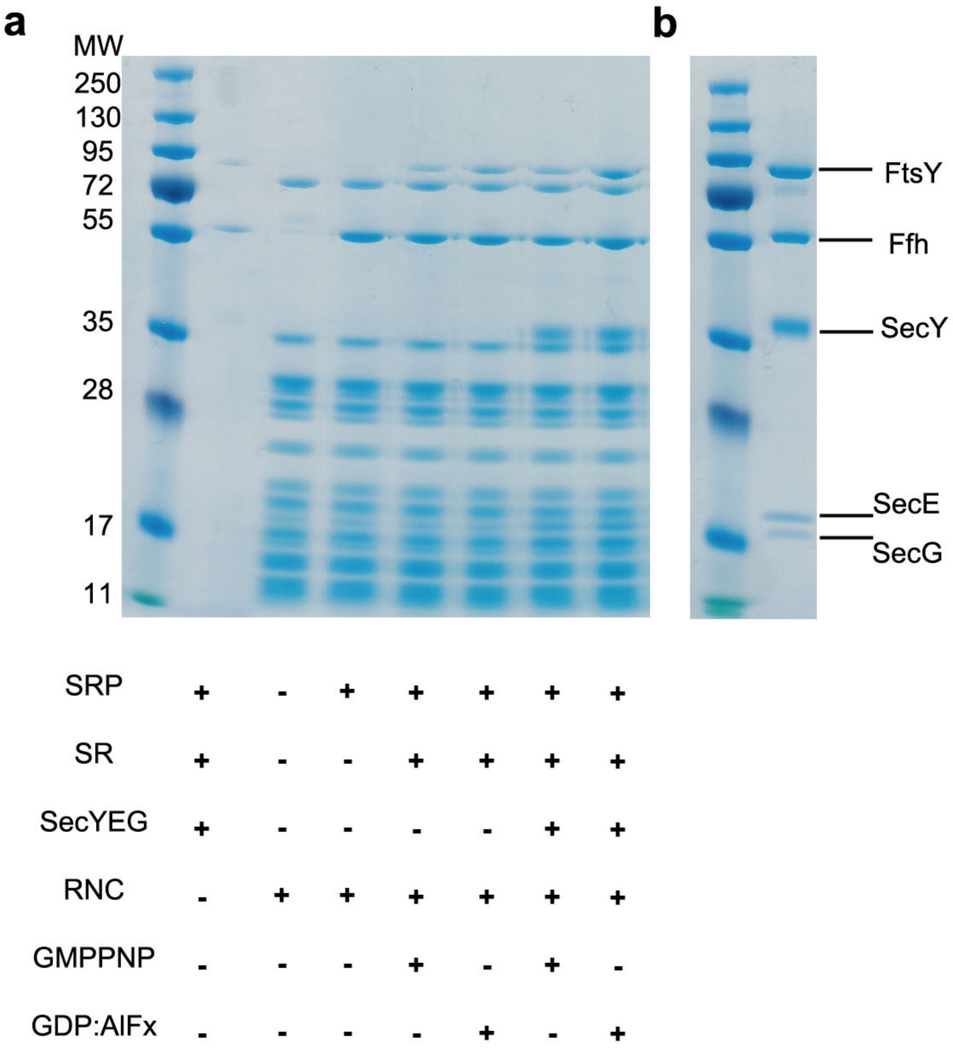


**Supplementary Figure 1: Assembly of the RNC-SRP-SR-Sec translocon quaternary complex.** Purified components were mixed in 1:5:5:5 molar rations as described (Material and Methods) and incubated for 30 minutes at 25 ° C in the presence of either GMPPNP or GDP:AlFx before applying the sample on a 40% (w/v) sucrose cushion and pelleting by ultra-centrifugation. **a**, Pellets were resuspended in Buffer B, resolved by 12% SDS PAGE, and stained by Comassie brilliant blue. **b**, Supernatant fraction of lane 2 in Panel **a** was resolved separately by SDS PAGE as a control. In the presence of GTP analogues (GMPPNP or GDP:AlFx), we observe stoichiometric binding of all components of the quaternary complex.

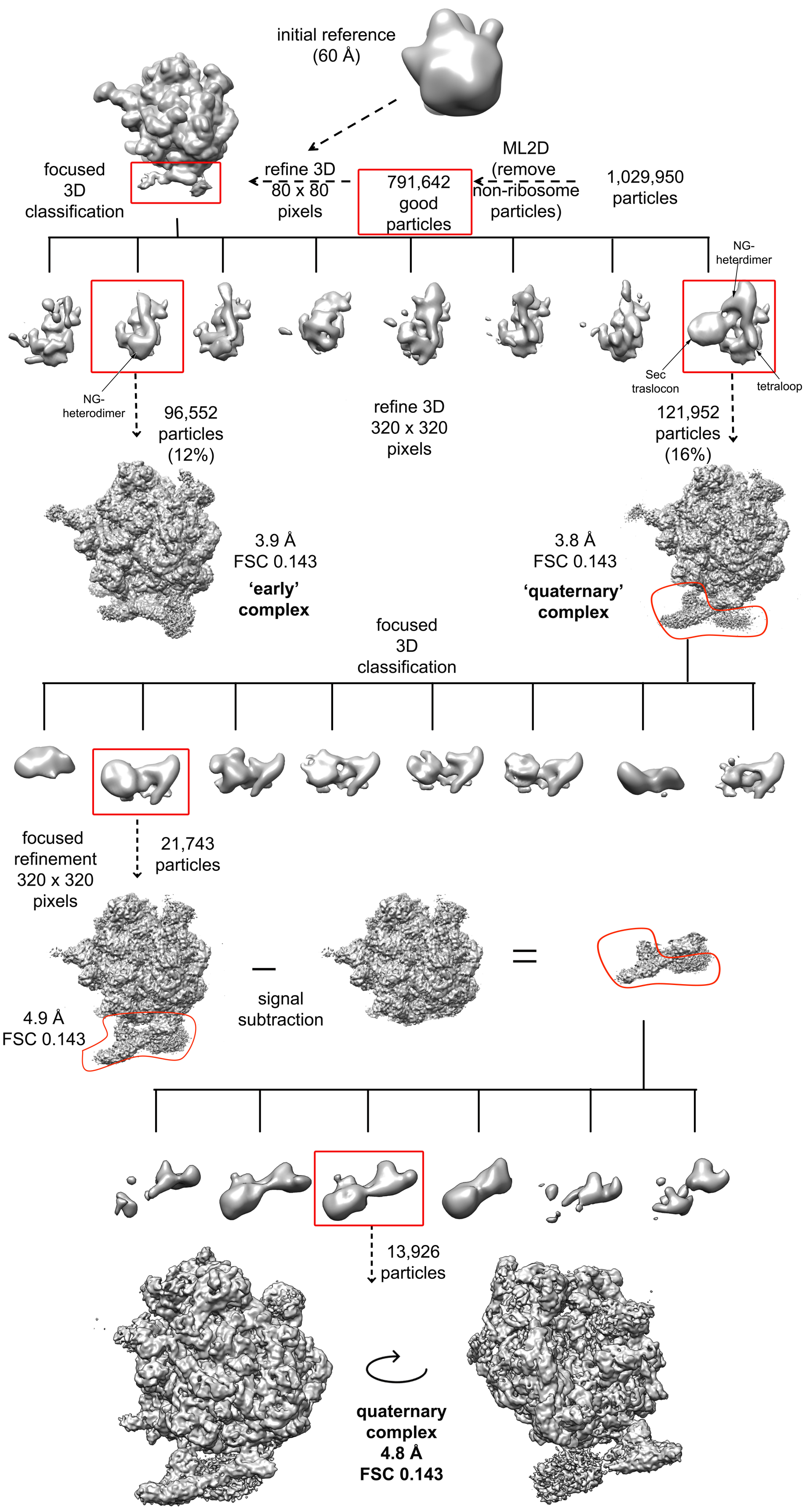
initial reference
(60 Å)
focused
3D
classification
refine 3D
80 x 80
pixels
791,642
good
particles
ML2D
(remove
non-ribosome
particles)
1,029,950
particles
NG-
heterdimer
Sec
traslocon
tetraloop
NG-
heterodimer
96,552
particles
(12%)
refine 3D
320 x 320
pixels
121,952
particles
(16%)
3.9 Å
FSC 0.143
'early'
complex
3.8 Å
FSC 0.143
'quaternary'
complex
focused
3D
classification
focused
refinement
320 x 320
pixels
21,743
particles
4.9 Å
FSC 0.143
signal
subtraction
13,926
particles
quaternary
complex
4.8 Å
FSC 0.143

**Supplementary Figure 2: 2D and 3D classification and refinement scheme.** A total of 1,029,950 particles of the reaction containing $RNC_{1A9L}$-85, SRP, SR, and Sec translocon assembled in the presence of GDP:AlFx were semi-automatically picked with BOXER in the EMAN package. Two-dimensional classification (ML2D) was performed for 30 iterations using RELION on 4-fold binned images (80x80 pixels) with a sampling of 5.56 Å/pixel. 791,642 particles from 'good' 2D class averages were retained and refined with an empty 70S model low-pass filtered to 60 Å resolution. Initial focused classification was performed for 30 iterations with the 'skip-align' option and by applying a circular mask on the polypeptide exit tunnel region. Selected classes were then refined using information up to Nyquist frequency with a sampling of 1.39 Å/pixel. 121,952 particles (16%) depicted a quaternary complex with the kinked RNA structure and a density for the detergent embedded translocon, and were refined to 3.8 Å resolution. To improve the density of the translocon, an additional round of focused classification using the 'skip-align' option was performed by applying a mask on the SRP distal region and the translocon and excluding the ribosome and the SRP M-domain regions. Further local classification was then performed using the signal subtraction approach implemented in RELION [1] by subtracting the signal of the ribosome. This was followed by a focused refinement to 4.8 Å of the particles by masking out the small ribosomal subunit. The resolution was determined based on the gold standard FSC (0.143 criterion).

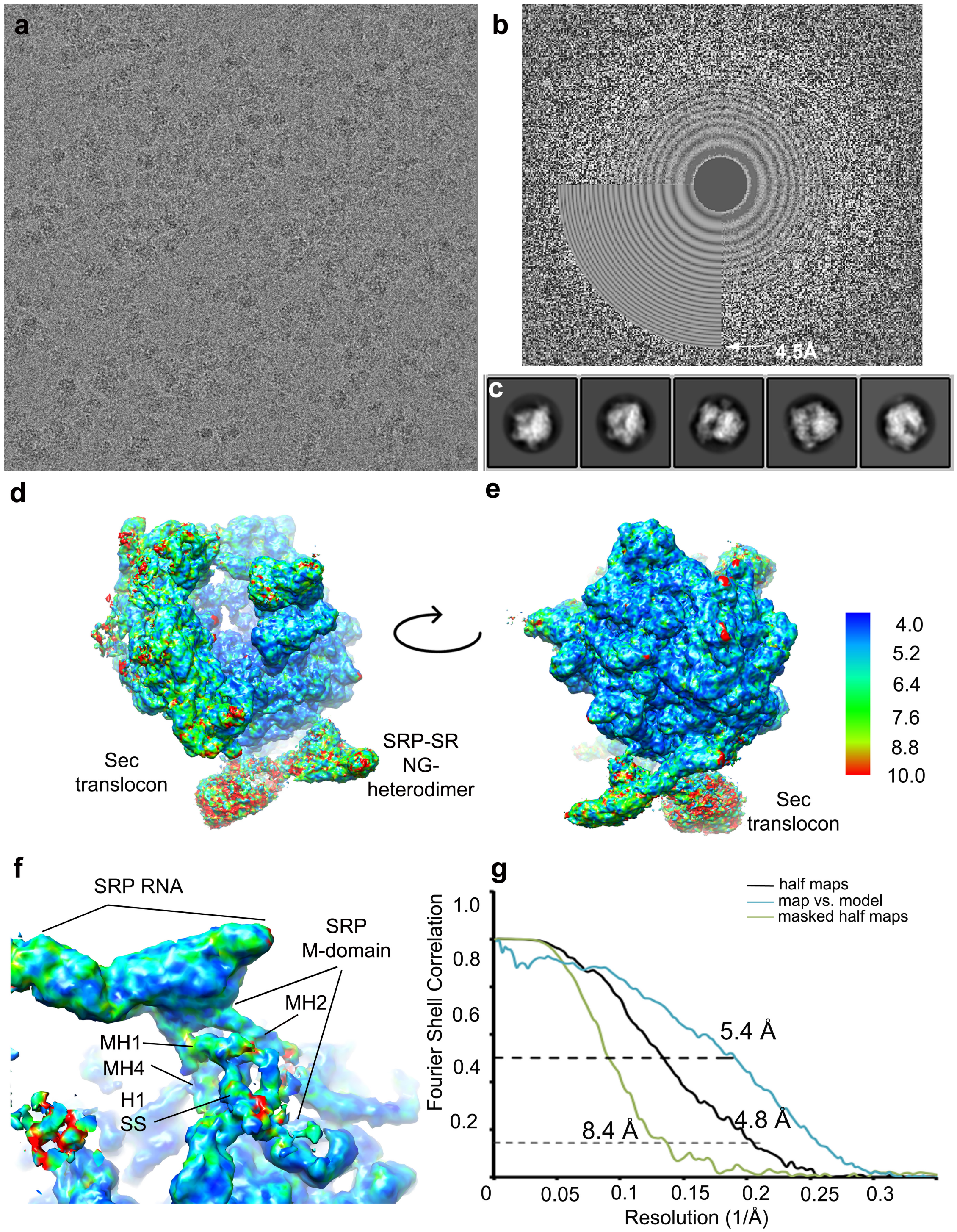
a
b
4.5Å
c
d
e
Sec
translocon
SRP-SR
NG-
heterodimer
4.0
5.2
6.4
7.6
8.8
10.0
Sec
translocon
f
SRP RNA
SRP
M-domain
MH2
MH1
MH4
H1
SS
g
half maps
map vs. model
masked half maps
Fourier Shell Correlation
1.0
0.8
0.6
0.4
0.2
5.4 Å
4.8 Å
8.4 Å
0
0.05
0.1
0.15
0.2
0.25
0.3
Resolution (1/Å)

**Supplementary Figure 3: Local resolution estimation and Fourier shell correlation plot of the cryo-EM map of the quaternary complex. a**, Representative cryo-EM micrograph of the assembled reaction containing SRP, SR, Sec translocon and RNC assembled in the presence of GDP:AlFx. **b**, Corresponding Thon rings extending to 4.5 Å resolution. **c**, Representative 2D class averages obtained after running 30 cycles of maximum-likelihood classification. **d** and **e**, Unsharpened cryo-EM map of the quaternary complex colored based on local resolution calculated using ResMap [2]. The resolution extends to better than 4 Å in the core of the ribosome. The local resolution of the NG-heterodimer is ~7 Å, of the M-domain ~5 Å, and of the Sec translocon ~7-10 Å. **f**, Close-up view of the SRP RNA and the M-domain (SRP core) bound to the ribosome with resolved protein $\alpha$ helices of the M-domain and the signal sequence (SS). **g**, Fourier shell correlation curves for the full two half maps (black), masked half maps by masking the area of the SRP-SR-Sec translocon and excluding the ribosome (green), and map versus model (blue) are shown. Resolutions of the full map and of the map area masked around the factors were estimated according to the gold standard FSC=0.143 criterion as implemented in RELION. The resolution of the model versus map was estimated based on the FSC=0.5 criterion.

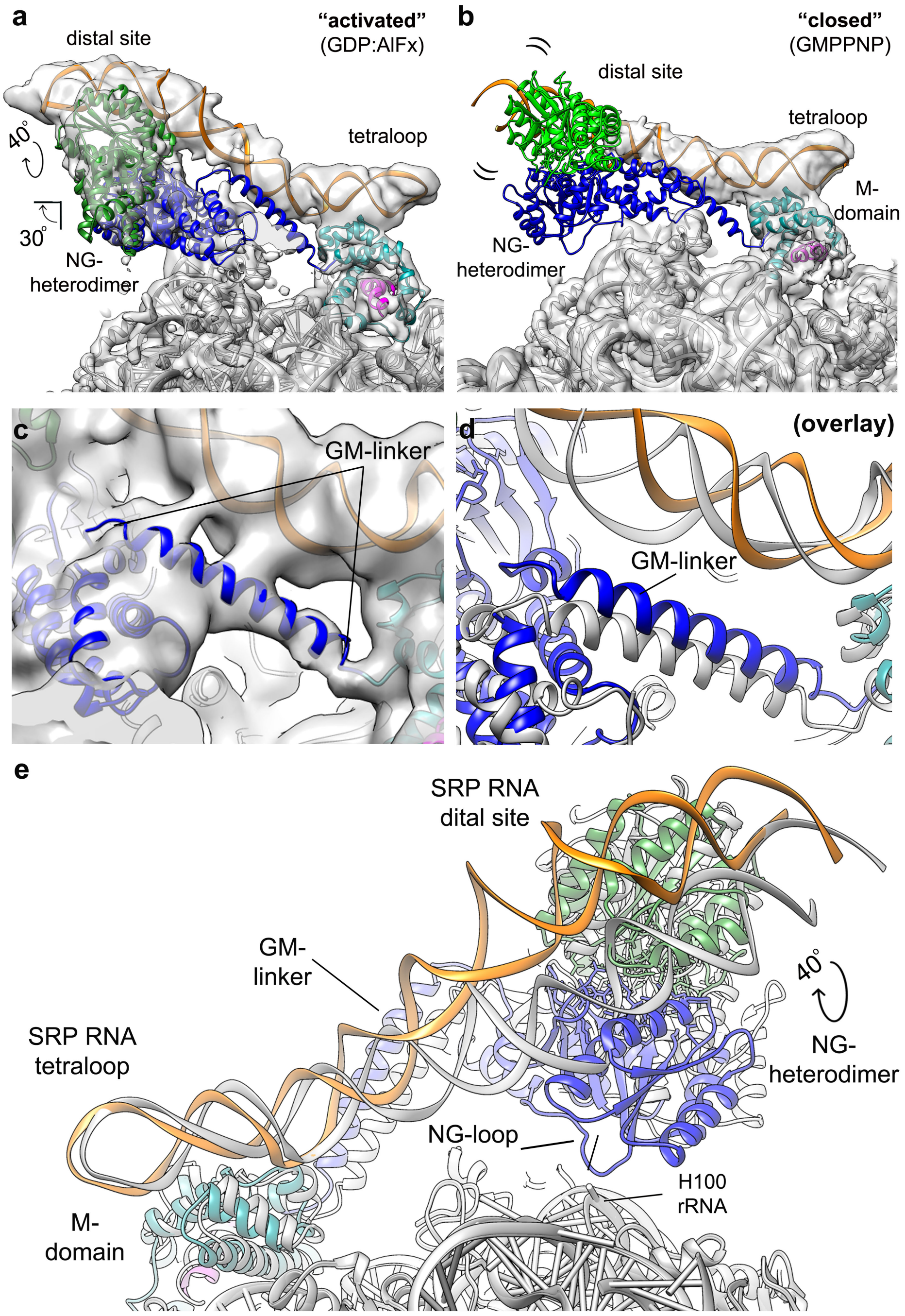


**f**

```
H.pylori          -mfqalsd-gfknalnkirfq--ddekaldraldelkktllkndvhhkvarellkkvesq   56
M.pneumoniae      -mfksmissivmrsmqkkinaqtisetdvqavlkeiriallDadvnllvvknfikaireq   59
E.coli            -mfdnltd-rlsrtlrnisgrgrltednvkdtlrevrmalleadvalpvvrefinrvkek   58
L.hackeliae       -mfenlte-rltrafktlsgqghlteenvhdalrevrlslleadvafpvvkefieevkqk   58
B.subtilis        mafeglad-rlqqtiskirgkgkvseqdvkdmmrevrlalleadvnfkvvkdfvkkvser   59
S.mutans          mafeslte-rlqgvfknlrgkrklsekdvqevtkeirlalleadvalpvvkefikrvrkr   59
                    *. : .  .   : .        *  :.    *:: :**. **   *.::::: : .:

H.pylori          tkls----gi-gkqqfldalekslleilsakgssgftfaqtpptvvlmaglqgsgktttt   111
M.pneumoniae      tvgqtvepgqdlqkwllkvikqelinilsq-pnqei--tskrplkvmvvglqgsgktttc   116
E.coli            avghevnksltpgqefvkivrnelvaamge-enqtlnlaaqppavvlmaglqgagkttsv   117
L.hackeliae       algqkvatnlkpeqafvkvvhdelvhvlgd-sraelnfktqppavflmaglqgsgkttss   117
B.subtilis        avgqdvmksltpgqqvikvvkeeltelmgg-eeskiavakrpptvimmvglqgagkttts   118
S.mutans          avghevidtldpsqqiikivneeltavlgs-etaeieksskiptiimmvglqgagkttfa   118
                  :            : .:. :...*   :.      :      *  .::.****:****
```

**Supplementary Figure 4: Conformation of the SRP RNA in the quaternary complex.** **a** and **b**, Close-up views of the SRP-SR NG heterodimer in the activated state solved in the presence of the transition state analogue (GDP:AlFx; this study) and the SRP-SR in the closed state solved in the presence of the non-hydrolysable GTP analogue (GMPPNP; EMDB-8003). Atomic model of the NG-heterodimer in Panel **b** was generated by docking the x-ray structure of SRP-SR at the distal site (PDB:2XXA) into the EM map (EMDB-8003) and by using the M-domain as a reference (PDB: 5GAG). **c**, Close-up view of the GM-linker region shown at lower contour level (1.5 σ). Cryo-EM densities were filtered to 6 Å for clarity. **d** and **e**, Overlay of the atomic models in Panels **a** and **b**. Coordinates of the closed complex are colored gray. **f**, Sequence alignment of the NG-loop depicts the sequence conservation in bacterial systems and was produced using the ClustalW WebServer [3,4].

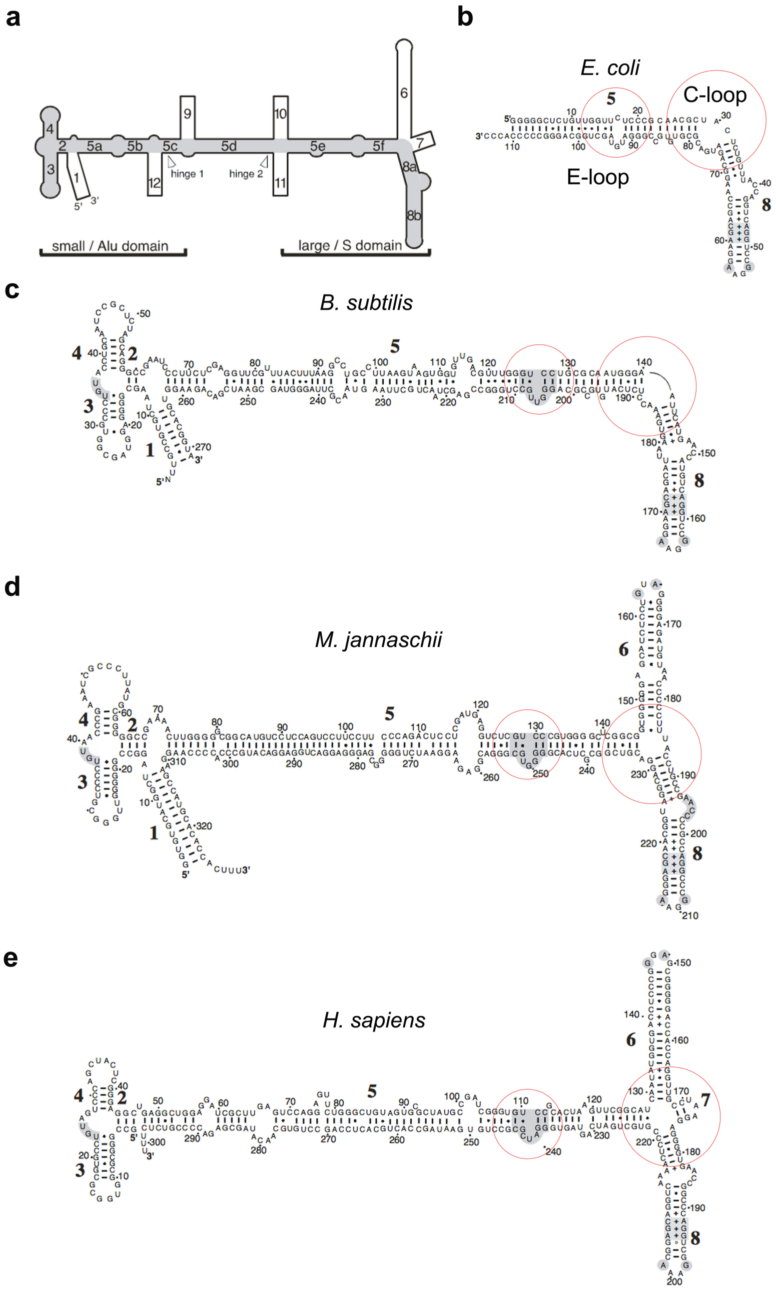


**Supplementary Figure 5**: **Secondary structure diagrams of SRP RNA from different organisms. a**, Schematic and nomenclature of all SRP RNAs [5]. Secondary structure of SRP RNA from **a**, *Escherichia coli*, **b**, *Bacillus subtilis,* **c**, *Methanocaldococcus jannaschi*, **d**, and *Homo sapiens*, **e**. RNA secondary structure diagrams were downloaded from the SRP database (http://bio.lundberg.gu.se/dbs/SRPDB/SRPDB.html). Circled regions indicate the hinge region observed in this study, also known as C- and E-loops, where the SRP RNA is kinked and untwisted, respectively.

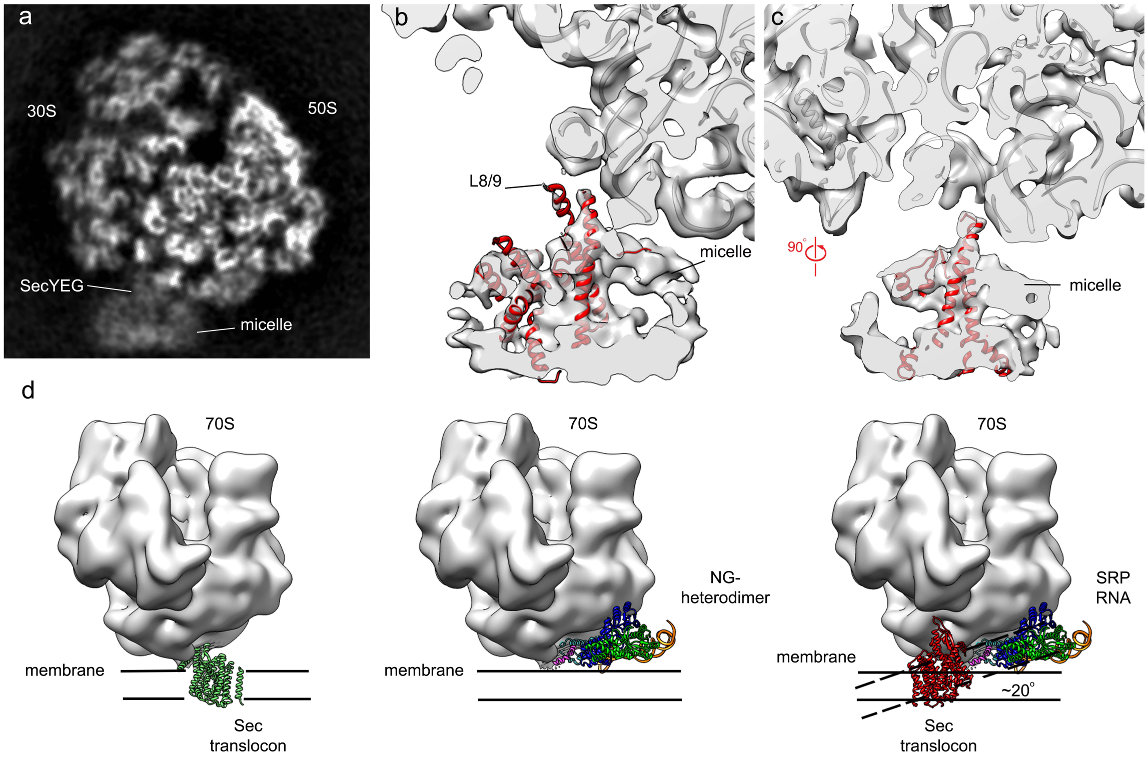


**Supplementary Figure 6: Orientation of the observed Sec translocon relative to the membrane.** **a**, Cross-section of the unsharpened EM-density map of the quaternary complex showing secondary structural elements of the Sec translocon within the unstructured detergent micelle. **b** and **c**, Cut-through snapshots of the Sec translocon into the EM-density filtered to 8 Å with the homology model based on the x-ray coordinates of the isolated SecYEG (PDB:5CH4) docked as a rigid body (red). **d**, The proposed 20° tilt in the position of the translocon to prevent clashes with SRP and SR. The plane of the membrane is shown schematically as resolved in the structures of the native Sec translocon [6] or in nano-discs [7] bound at the ribosomal exit tunnel.

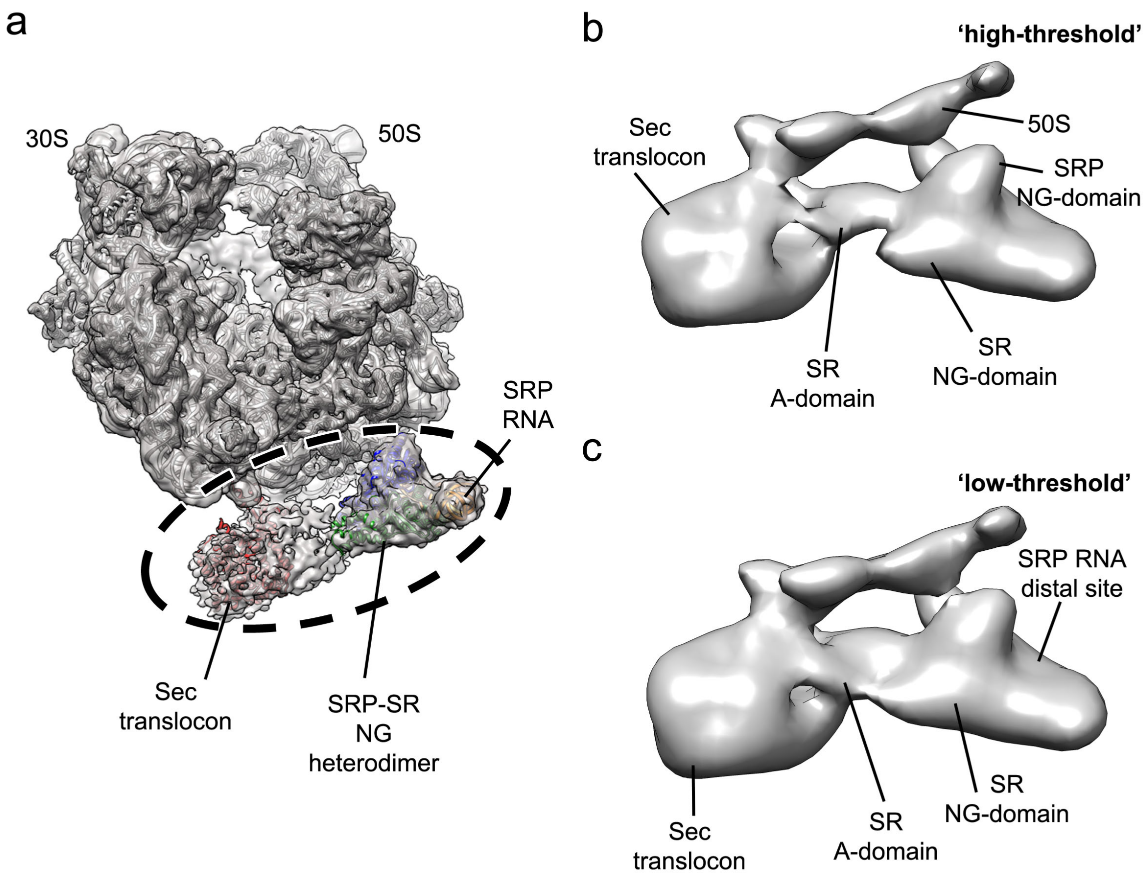


**Supplementary Figure 7: Position of the A-domain of SR in the quaternary complex.** **a**, Snapshot of the cryo-EM density of the quaternary complex with fitted atomic coordinates. **b** and **c**, Close-up view of the circled area in Panel **a**, filtered to 20 Å and displayed at high and low density thresholds. Densities of the Sec translocon, SRP, and A-domain and NG-domain of SR are indicated. The surface of the ribosome is removed for clarity.

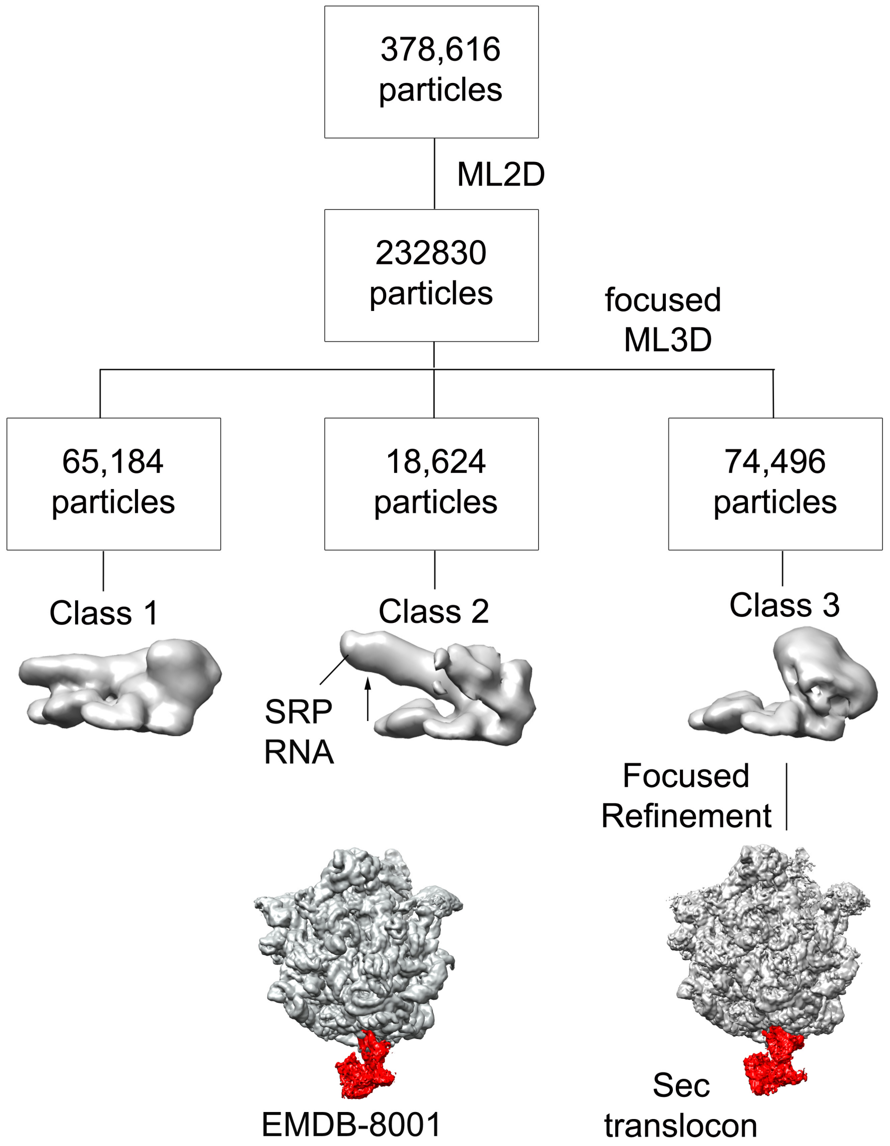


**Supplementary Figure 8**: **Cryo-EM analysis of the $RNC_{1A9L}$-135 in the presence of the SRP, SR, Sec translocon and GDP:AlFx.** The dataset was subjected to a similar processing approach as described in Extended Data Figure 2. Only subclasses of the focused 3D classification with recognizable features of either SRP, SR, or Sec translocon are shown. EMDB-8001 is filtered to a similar resolution as the final reconstruction for comparison.

| DATA COLLECTION | 50S-SRP-SR-SecYEG (SRP-SR-SecYEG) |
|---|---|
| Final number of Particles | 13,926 |
| Pixel size (Å) | 1.39 |
| Defocus range (µm) | 1.2 - 3.6 |
| Voltage (kV) | 300 |
| Electron does ($e^-$ $Å^{-2}$) | 20 |
| **Model Refinement** | |
| **Refinement** | |
| Resolution (Å) | 4.8 (8.4) |
| Map Sharpening B-factor ($Å^2$) | -218 |
| Average B-factor ($Å^2$) | 280 |
| **R.m.s deviations** | |
| Bond length (Å) | 0.007 |
| Bond angles (°) | 1.073 |
| **VALIDATION STATISTICS** | |
| **Validation** | |
| Molprobity Score | 2.35 (2.53) |
| Clashscore, all atoms | 8.37 (16.6) |
| Good rotamers (%) | 91.5 (91.3) |
| **Ramachandran Plot** | |
| Favored (%) | 96.8 (94.7) |
| Allowed (%) | 3.1 (4.44) |
| Outliers(%) | 0.08 (0.86) |
| **Validation (RNA)** | |
| Correct sugar puckers (%) | 99.5 (98.1) |
| Good backbone conformation (%) | 82.2 (71.2) |

**Supplementary Table 1.** Model and refinement statistics

| COMPONENT | CHAIN ID | SOURCE | COMMENT |
| --- | --- | --- | --- |
| SRP RNA (distal site) | 1 | PDB ID 4C7O | Fitted as a rigid model |
| SRP RNA (core) | 1 | PDB ID 5GAG | Fitted as a rigid model |
| SRP RNA (kinked region) (res. 28:44, 70:80) | 1 | - | Modeled |
| Ffh (M-domain) | i | PDB ID 5GAG | Fitted as a rigid model |
| Ffh (NG-domain) | i | PDB ID 4C7O | Fitted as a rigid model |
| Ffh (GM-linker) | i | PDB ID 2XXA | Fitted as a rigid model |
| FtsY (NG-domain) | l | PDB ID 4C7O | Fitted as a rigid model |
| Signal sequence | k | - | Modeled |
| SecY | g | PDB ID 5CH4 | Homology model |
| SecE | h | PDB ID 5CH4 | Homology model |
| SecG | j | PDB ID 5CH4 | Homology model |
| 50S | - | PDB ID 5GAG | Fitted as a rigid body |

**Supplementary Table 2.** Components of the final model used for rigid body docking and modeling

| Region | Chain ID | Resolution (ResMap) |
|---|---|---|
| SRP protein M-domain | i (res. 329-453) | 6.5 |
| Signal sequence | k | 6.7 |
| SRP protein NG-domain | i (res. 1-328) | 8.0 |
| SRP RNA | 1 | 8.0 |
| SR NG-domain | l | 9.2 |
| SecYEG | g,h,j | 9.5 |

**Supplementary Table 3.** Local-resolution based on the atom position of the fitted model

**References**

1. Bai, X.C., Rajendra, E., Yang, G., Shi, Y. & Scheres, S.H. Sampling the conformational space of the catalytic subunit of human gamma-secretase. *Elife* **4**(2015).
2. Kucukelbir, A., Sigworth, F.J. & Tagare, H.D. Quantifying the local resolution of cryo-EM density maps. *Nat Methods* **11**, 63-5 (2014).
3. Larkin, M.A. et al. Clustal W and Clustal X version 2.0. *Bioinformatics* **23**, 2947-8 (2007).
4. Goujon, M. et al. A new bioinformatics analysis tools framework at EMBL-EBI. *Nucleic Acids Res* **38**, W695-9 (2010).
5. Zwieb, C., van Nues, R.W., Rosenblad, M.A., Brown, J.D. & Samuelsson, T. A nomenclature for all signal recognition particle RNAs. *RNA* **11**, 7-13 (2005).
6. Pfeffer, S. et al. Structure of the native Sec61 protein-conducting channel. *Nat Commun* **6**, 8403 (2015).
7. Frauenfeld, J. et al. Cryo-EM structure of the ribosome-SecYE complex in the membrane environment. *Nat Struct Mol Biol* **18**, 614-21 (2011).
